# Editorial: Machine learning and signal processing for neurotechnologies and brain-computer interactions out of the lab

**DOI:** 10.3389/fnrgo.2023.1305482

**Published:** 2023-11-09

**Authors:** Mahnaz Arvaneh, Toshihisa Tanaka, Haider Raza, Masaki Nakanishi, Tomas Emmanuel Ward

**Affiliations:** ^1^Automatic Control and Systems Engineering, University of Sheffield, Sheffield, United Kingdom; ^2^Tokyo University of Agriculture and Technology, Tokyo, Japan; ^3^School of Computer Science and Electronics Engineering, University of Essex, Colchester, United Kingdom; ^4^Institute for Neural Computation, University of California, San Diego, La Jolla, CA, United States; ^5^Insight SFI Research Centre for Data Analytics, Dublin, Ireland

**Keywords:** brain computer interface, machine learning, neurotechnologies, signal processing, neuroscience

The merging of neuroscience with technology gives rise to Brain-Computer Interfaces (BCIs), a groundbreaking domain driving innovation in signal processing, machine learning, biomedical instrumentation and wearable sensors. BCIs (Vidal, 1973) hold the promise of direct mind-machine communication, offering empowerment to individuals with paralysis, and augmenting cognitive abilities, and are in the vanguard of a new era of human-computer symbiosis.

This Research Topic aims to present the latest research findings in Machine Learning and Signal Processing for neurotechnologies. It explores progress toward transitioning this technology from controlled laboratory and clinical settings to real-world applications, often referred to as “in the wild” deployments. By enabling real-world applications, BCIs can enhance accessibility for various fields, including healthcare and assistive technology, unlocking the potential for widespread societal benefits and fostering innovation beyond controlled settings (Pirondini et al., 2017). The pursuit of new and improved machine learning and signal processing for neurotechnologies attracts a lot of new researchers as it presents very challenging problems which require innovative solutions and transdisciplinary approaches. Extending these technologies beyond the laboratory context into real-world applications promises groundbreaking new solution areas for BCIs. This fusion holds the key to enhancing lives through accessible healthcare, assistive devices, and augmented human capabilities, inspiring us to contribute to this transformative frontier.

This editorial topic discusses four significant research papers, each contributing to the advancement of BCIs and signal denoising techniques for real-world applications, which may lead to bringing BCIs out of the lab in the near future.

Pham Xuan et al. explore the potential use of Passive Brain-Computer Interfaces (pBCIs) to detect cognitive processes related to face recognition. The aim is to apply this technology in autonomous vehicles to interpret facial mimicry and non-verbal communication in complex real-world scenarios. The pBCI achieved an accuracy of over 70% in distinguishing responses to faces from other stimuli in a single trial, demonstrating promise for enhancing safety and communication in autonomous vehicles.

Brophy et al. address the challenge of noise in EEG signals, which is crucial for BCIs. The study employs Generative Adversarial Networks (GANs) to denoise EEG data, enhancing BCI performance in practical settings. This GAN-based approach offers versatility, making it adaptable to low-resource scenarios. The research validates the effectiveness of GANs in EEG artefact removal, advancing the state-of-the-art in EEG signal enhancement. This has promising implications for more accurate and stable brain signal measurements in BCIs.

Giles et al. introduce a transfer learning algorithm, r-KLwDSA, designed to reduce the calibration time required for motor imagery-based BCIs, particularly for long-term users. The algorithm aligns EEG data from previous sessions with the current session, leading to a significant enhancement in classification accuracy, especially for sessions initially below 60%. This development has implications for improving BCI rehabilitation, particularly for stroke patients. Additionally, it addresses the challenge of domain adaptation in non-stationary settings, which may help in bringing BCI out of the lab.

Awais et al. presents the AMBER dataset, designed to facilitate the development of signal-denoising techniques for EEG measurements in real-world settings. The AMBER dataset combines EEG recordings with synchronized video data, enhancing analysis with rich contextual cues such as facial expressions and body movements, enriching research insights. This dataset is a significant advancement in BCI research, addressing the critical issue of signal denoising in EEG data, and serves as a valuable resource for exploring and advancing signal-denoising techniques, enhancing the reliability of BCIs in real-world environments.

In summary, these studies collectively contribute to the practicality and efficiency of BCIs in real-world applications, ranging from autonomous vehicles to rehabilitation and signal denoising, ultimately improving the quality and reliability of brain signal measurements and enhancing the potential of BCIs in various domains.

## Author contributions

HR: Writing—original draft, Writing—review and editing. MA: Writing—original draft, Writing—review and editing. TT: Writing—original draft, Writing—review and editing. MN: Writing—review and editing. TW: Writing—original draft, Writing—review and editing.
